# Visceral artery aneurysms

**DOI:** 10.1007/s00772-018-0384-x

**Published:** 2018-04-20

**Authors:** B. Juntermanns, J. Bernheim, K. Karaindros, M. Walensi, J. N. Hoffmann

**Affiliations:** 1 Department of Vascular Surgery and Phlebology, Contilia Heart and Vascular Center, Elisabeth Hospital, Klara-Kopp-Weg 1, 45138 Essen, Germany; 2 Practice for Vascular Surgery and Phlebology, Contilia Heart and Vascular Center, Essen, Germany

**Keywords:** Visceral artery, Aneurysm rupture, Endovascular therapy, Open surgical therapy, Indication, Viszeralarterien, Aneurysmaruptur, Endovaskuläre Therapie, Offen-chirurgische Therapie, Indikation

## Abstract

Visceral artery aneurysms are rare with an incidence of only 0.01–0.1% of the population. Open surgical or endovascular elimination should be performed for aneurysms greater than 2 cm in size. The risk of aneurysm rupture is then approximately 25–40%. If the aneurysm ruptures the mortality can be as high as 76%. For mycotic aneurysms or spurious aneurysms there is no lower limit to the diameter size for the need of treatment. Sudden abdominal pain during pregnancy can be caused by visceral artery aneurysms and must be further clarified. The indications for surgery during pregnancy should be made generously. The clinical symptoms (abdominal complaints) of visceral artery aneurysms are manifold. The treatment can be either an open surgical approach or endovascular treatment. In the emergency setting, if endovascular treatment is no longer possible, an open surgical treatment needs to be performed. There are so far no randomized studies which could identify one of the procedures (open surgery vs. endovascular surgery) as clearly being superior. The prognosis after treatment is satisfactory with a 5–10 year survival rate of approximately 90%.

## Zusammenfassung

Viszeralarterienaneurysmen sind mit nur etwa 0,01–0,1 % in der Bevölkerung eine Rarität. Die offen-chirurgische oder auch endovaskuläre Ausschaltung sollte ab einer Aneurysmagröße von 2 cm durchgeführt werden. Das Rupturrisiko der Aneurysmen liegt dann bei 25–40 %. Kommt es zur Ruptur, liegt die Letalität bei bis zu 76 %. Bei mykotischen Aneurysmen oder Aneurysma spuria gibt es keine Untergrenze des Durchmessers zur Indikationsstellung. Plötzlich auftretende abdominelle Schmerzen in der Schwangerschaft können durch ein Viszeralarterienaneurysma bedingt sein und müssen unbedingt abgeklärt werden. Die Indikation zur Therapie in der Schwangerschaft sollte großzügig gestellt werden. Die klinischen Symptome (abdominelle Beschwerden) der Viszeralarterienaneurysmen sind mannigfaltig. In der Notfallsituation sollte, wenn die endovaskuläre Therapie nicht mehr möglich ist, die offen-chirurgische Versorgung erfolgen. Es existieren bisher keine randomisierten Studien, die ein Verfahren (offen-chirurgisch vs. endovaskulär) eindeutig als überlegen identifizieren konnten. Die Prognose nach Versorgung ist gut mit Fünf- bis Zehnjahresüberlebensraten von rund 90 %.

## Background

Visceral artery aneurysms are rare with an incidence of approximately 0.01–0.1% in the population [1]. Aneurysms of the splenic artery are the most common, followed by the hepatic artery and the superior and inferior mesenteric arteries [2]. The distribution of visceral artery aneurysms is as follows [3]:

- Splenic artery aneurysm (60%)
- Hepatic artery aneurysm (20–50%)
- Superior mesenteric artery aneurysm (6%)
- Celiac artery aneurysm (4%)
- Spurious aneurysm (rare)
- Mycotic aneurysm (rare)

There are no gender differences in the distribution of aneurysms. Visceral artery aneurysms are often caused by pre-existing arteriosclerotic damage to vessel walls (70–90%), with older patients being particularly affected. Fibromuscular dysplasia is occasionally causative in younger patients. Rarer causes include mycosis (ca. 12.9%), giant cell arteritis (3.2%) and gene defects, such as those in Marfan or Ehlers-Danlos syndrome. The highest rupture rate is seen in hepatic artery aneurysms (ca. 80%) followed by pancreaticoduodenal artery aneurysms (75%) [4]. Inflammatory visceral artery aneurysms, which are often associated with a nearby infectious process (e. g., pancreatitis, pancreatic fistula), represent a special aneurysm type that poses a particular risk due to its rapid growth [5]. Traumatic aneurysms following, e. g., traffic accidents, as well as iatrogenic aneurysms following interventional/surgical treatment have also been described [4, 6–8].

## Visceral artery aneurysms during pregnancy

The highest rate of visceral artery aneurysms is seen in young expectant mothers and multiparous women [4]. Spontaneous rupture of a visceral artery aneurysm is a serious and potentially life-threatening complication during pregnancy, both for the expectant mother and the unborn child. The maternal mortality rate is 70–75%, while the mortality rate for the unborn child is 90–95% [9]. The pathomechanisms of aneurysm formation during pregnancy are still unclear; various theories postulate primarily hemodynamic and hormonal changes in the late stages of pregnancy. Surprisingly, it has been reported that almost 100% of all superior mesenteric artery aneurysms and up to 40% of splenic artery aneurysms rupture during pregnancy. The last trimester and the early phase following birth are particularly hazardous. Between 24% and 45% of visceral artery aneurysms rupture during this period [4]; however, rupture in the first trimester of pregnancy has also been described in individual case reports [10, 11]. Therefore, the indications to treat all visceral artery aneurysms diagnosed during pregnancy, most of which are incidental findings on ultrasound, should be made generously [4].

## Pathophysiology and clinical presentation

**Fig. 1:**
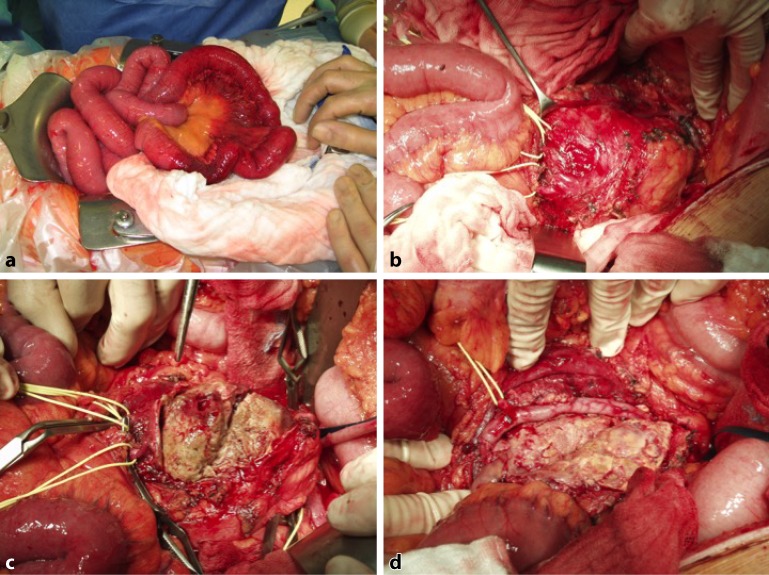
Macroscopic views of a large mesenteric artery aneurysm with peripheral embolization and the clinical picture of acute abdomen. **a** Partial small bowel infarction. **b** A large aneurysm of the superior mesenteric artery. The afferent and efferent side is visualized and accordingly clamped. **c** Once opened, the typical arteriosclerotic aneurysm content is seen. **d** Superior mesenteric artery repair using a reversed greater saphenous vein interposition graft. No partial resection of the ischemic parts of the small intestine was subsequently required since the intestine recovered completely

Small visceral artery aneurysms tend to be asymptomatic. Not until a diameter of 2 cm has been reached can the onset of pathophysiological complications be seen, such as aneurysm rupture, peripheral embolization, thrombotic occlusion of the aneurysmal vessel, and compression of neighboring structures [2]. Besides general clinical symptoms such as abdominal pain, full-blown acute abdomen, and signs of shock and sepsis, symptoms depend on the function of the respective end organ supplied by the aneurysmal vessel. Visceral artery aneurysms measuring over 2 cm in diameter are highly susceptible to rupture, having a rupture risk of 25–40% and a mortality rate after rupture of up to 76% [12, 13]. From a clinical perspective, loss of organ function and signs of hemorrhage predominate in the case of rupture. Ischemia may develop in the affected end organ in the event of peripheral embolization of thrombi (Fig. 1) that have formed in afferent aneurysmal vessels. Symptoms vary depending on the organ and lead to ischemia-related symptoms. In the case of thrombotic occlusion of the major intestinal vessels, chronic progressive ischemia of the respective organs can be seen. Clinical manifestations range from abdominal angina to acute mesenteric infarction. Large aneurysms of the celiac artery and its branches can cause compression of neighboring structures such as the stomach, small intestine, and even the bile ducts, resulting in upper abdominal pain, cholestasis, episodes of jaundice, and impaired intestinal passage [2]. Gastrointestinal bleeding as a sign of aneurysm perforation in the respective neighboring hollow organs is possible [14].

## Diagnosis

As mentioned many visceral artery aneurysms are asymptomatic and are detected as a result of the growing availability of cross-sectional imaging; however, visceral artery aneurysm is also frequently diagnosed as an incidental finding on abdominal ultrasound or X‑ray examination based on the detection of crescent-shaped calcification in the aneurysm [2]. On Doppler duplex ultrasound, visceral artery aneurysms appear as round, hypoechoic, and, depending on the size of the rim of the thrombus, perfused masses [4]. Treatment planning should ideally include cross-sectional imaging using computed tomography (CT) or magnetic resonance (MR) angiography [4].

## Indications and treatment

The indications for elective treatment of visceral artery aneurysm are made from a diameter of 2 cm. Multiple hepatic artery aneurysms, which are not caused by arteriosclerosis and show no symptoms, are an exception here. As such, these aneurysms should be repaired from a diameter of <2 cm due to an increased risk of rupture [4]. Similarly, in the case of mycotic or spurious aneurysms, there is no lower diameter threshold for treatment: interventional or surgical elimination is always indicated [4]. Mortality rises to 25% and higher in the case of treatment in an emergency setting [15]. The German Society for Vascular Surgery (Deutsche Gesellschaft für Gefäßchirurgie, DGG) guidelines [2] provide a detailed overview of the open procedure for aneurysm elimination/repair: They state the following: in the case of surgical elimination of a visceral artery aneurysm, localization and potential collateral circulation to the end organ need to be taken into consideration. The goal is to maintain perfusion to ischemia-sensitive end organs (splenic artery aneurysm is an exception, since artery coiling is possible here). The access route most commonly used is midline laparotomy, which also offers a good overview of retroperitoneal vascular structures. In the case of aneurysms of the large celiac artery or superior mesenteric artery, left-sided or right-sided medial visceral rotation according to Kocher is helpful. Celiac aneurysms can also be well visualized using a transverse epigastric incision. In rare cases, thoracoabdominal access according to Crawford may be necessary. If collateral circulation is present, proximal and distal aneurysm ligature (with either removing or leaving the aneurysm itself) can be considered in aneurysms of the splenic artery, the proximal hepatic artery, the gastroduodenal artery, and the midline inferior mesenteric artery. If the afferent and efferent vessels are long, aneurysm resection and subsequent end-to-end anastomosis of the healthy vascular stumps in the sense of a continuity resection can be considered. It is absolutely crucial to avoid anastomotic tension here. In the case of large aneurysms, tension-free interposition grafts/bypasses can be inserted using a conventional inlay technique. If it is not possible to securely clamp the aneurysm, transaneurysmal catheter-mediated blockade can be considered. Although autologous veins are the material of first choice, short segment synthetic grafts are able to achieve equally good results. Shunt procedures have proven their worth in the reduction of intestinal ischemia time. Aneurysms that are essential in the distribution zones for end organs can be treated by aneurysmorrhaphy, i. e., aneurysm resection and subsequent wall repair. Spontaneous thrombosis and recurrent ectasia are rarely seen following this procedure. In the case of intraorgan aneurysms and no possibility of embolization, organ resections, such as partial liver resection, splenectomy, or pancreatic tail resection, are necessary. If the aneurysm is located in the pancreatic head, a Whipple procedure may also be required.

## Endovascular eliminaton

Due to the considerable surgical trauma associated with a conventional open procedure and a surgical mortality rate that is not to be underestimated, the endovascular treatment of visceral artery aneurysms has significantly gained in importance [16, 17]. Since attempts at embolization are hazardous due to the possibility of particle spread and are unable to completely eliminate the risk of rupture [14, 19, 20], covered stents or stent grafts are the treatment of first choice [14, 19, 20]. Nevertheless, embolization is an established and effective method in splenic artery aneurysms [18].

## Emergency surgery for ruptured visceral artery aneurysms

If an aneurysm ruptures and an endovascular approach is no longer possible, emergency open surgical treatment is necessary [21, 22]. In order to control the life-threatening bleeding that frequently occurs, the subphrenic aorta and, if necessary, the hepatoduodenal ligament should be clamped. Intraoperative angiography is indicated if it is not possible to adequately determine the cause of bleeding. Following clip ligation and hematoma evacuation, it is generally possible to treat an aneurysm while taking ischemia time into consideration. If it is not possible to preserve the superior mesenteric vein, venous reconstruction, where possible, is required in order to prevent mesenteric venous thrombosis [2]. The most serious complication of aneurysm elimination is ischemic tissue necrosis in end organs [23]. Postoperative intensive care monitoring is required. A second-look procedure should be carried out if ischemic complications are suspected. Late complications following successful endovascular or surgical repair are rare.

## Open or endovascular repair?

With respect to the superiority of the approaches in question, there are currently no randomized trials favoring one approach over the other [1]; however, the literature suggests that older patients in particular benefit from endovascular repair in the elective setting. Given the regular radiological follow-up examinations required following endovascular repair, younger patients should be treated using an open approach where possible. Open repair appears to be the better approach in the case of rupture [24]. Due to the considerable surgical trauma associated with open procedures, as well as the surgical mortality rate of approximately 1–3%, the DGG guidelines on the management of visceral artery aneurysms favor endovascular elimination using stent grafts or covered stents.

## Prognosis

Patients are considered cured following endovascular or surgical elimination of a visceral artery aneurysm. Long-term anticoagulation does not appear to be necessary. The 5 and 10-year survival rates following elimination are approximately 90% [2]. Imaging is not necessarily required following open repair of visceral artery aneurysms [4]. The rate of aneurysm revascularization following endovascular repair has been put at 35% [25], making regular cross-sectional imaging follow-up necessary. Regular CT angiography follow-up is only indicated in known aneurysmosis; however, the DGG guidelines recommend at least regular ultrasound follow-up [2]. Contrast-enhanced ultrasound, which is finding increasing application in the follow-up of endovascular aortic aneurysms, has not yet been included in the current DGG guidelines. In the authors’ opinion, this method for the follow-up of visceral artery aneurysm repair needs to be evaluated.

## Conclusion

- Visceral artery aneurysms are rare.
- Surgical/endovascular elimination should be performed from an aneurysm size of 2 cm, in which case the risk of rupture is 25–40%.
- In the case of rupture, the mortality rate is as high as 76%.
- There is no lower diameter threshold for treatment in mycotic or spurious aneurysms.
- Abdominal pain of sudden-onset during pregnancy can be caused by a visceral artery aneurysm and always requires investigation.
- The clinical (abdominal) symptoms of visceral artery aneurysm are manifold.
- Treatment can be performed using an open or an endovascular approach. To date, there are no randomized studies that have been able to identify one approach as being superior to the other.
- The prognosis following treatment is good, with 5 to 10-year survival rates of approximately 90%.

## References

[1] Debus S, Grundmann T (2015). Evidenzbasierte Gefäßchirurgie.

[2] Deutsche Gesellschaft für Gefäßchirurgie (2011). Leitlinien der Deutschen Gesellschaft für Gefäßchirurgie (vaskuläre und endovaskuläre Chirurgie) (DGG).

[3] Croner RS, Anders K, Uder M, Lang W (2006). Aneurysmen viszeraler Arterien. Dtsch Arztebl.

[4] Meyer A, Uder M, Lang W, Croner R (2010). Visceral artery aneurysms. Zentralbl Chir.

[5] Grotemeyer D, Grabitz K, Balzer K, Reinecke P, Poll L, Sandmann W (2004). Das mykotische Viszeralarterienaneurysma. Chirurg.

[6] Gen S, Usui R, Sasaki T, Nobe K, Takahashi A, Okudaira K, Ikeda N (2016). Infected aneurysm after endoscopic submucosal dissection. Intern Med.

[7] Vernadakis S, Christodoulou E, Treckmann J, Saner F, Paul A, Mathe Z (2009). Pseudoaneurysmal rupture of the common hepatic artery into the biliodigestive anastomosis. A rare cause of gastrointestinal bleeding. JOP.

[8] Pinsky MA, May ES, Taxier MS, Blackford J (1987). Late manifestation of hepatic artery pseudoaneurysm: case presentation and review. Am J Gastroenterol.

[9] Brunner E, Drewitz A, Dietz H, Rieger L (2015). Ruptur eines Milzarterienaneurysmas in der Schwangerschaft – Eine Kasuistik. Geburtshilfe Frauenheilkd.

[10] Groussolles M, Merveille M, Alacoque X, Vayssiere C, Reme JM, Parant O (2011). Rupture of a splenic artery aneurysm in the first trimester of pregnancy. J Emerg Med.

[11] Chookun J, Bounes V, Ducassé JL, Fourcade O (2009). Rupture of splenic artery aneurysm during early pregnancy: a rare and catastrophic event. Am J Emerg Med.

[12] Sadat U, Dar O, Walsh S, Varty K (2008). Splenic artery aneurysms in pregnancy – a systematic review. Int J Surg.

[13] Teng W, Sarfati MR, Mueller MT, Kraiss LW (2006). A ruptured pancreaticoduodenal artery aneurysm repaired by combined endovascular and open techniques. Ann Vasc Surg.

[14] Chiesa R, Astore D, Guzzo G (2005). Visceral artery aneurysms. Ann Vasc Surg.

[15] Ruiz-Tovar J, Martínez-Molina E, Morales V, Sanjuanbenito A, Lobo E (2007). Evolution of the therapeutic approach of visceral artery aneurysms. Scand J Surg.

[16] Jimenez JC, Lawrence PF, Reil TD (2008). Endovascular exclusion of superior mesenteric artery pseudoaneurysms: an alternative to open laparotomy in highrisk patients. Vasc Endovascular Surg.

[17] Carroccio A, Jacobs TS, Faries P, Carroccio A, Jacobs TS, Faries P, Ellozy SH, Teodorescu VJ, Ting W, Marin ML (2007). Endovascular treatment of visceral artery aneurysms. Vasc Endovascular Surg.

[18] Ikeda O, Tamura Y, Nakasone Y, Iryou Y, Yamashita Y (2008). Nonoperative management of unruptured visceral artery aneurysms: treatment by transcatheter coil embolization. J Vasc Surg.

[19] Rossi M, Rebonato A, Greco L, Citone M, David V (2008). Endovascular exclusion of visceral artery aneurysms with stent-grafts: technique and longterm follow-up. Cardiovasc Intervent Radiol.

[20] Tulsyan N, Kashyap VS, Greenberg RK, Sarac TP, Clair DG, Pierce G, Ouriel K (2007). The endovascular management of visceral artery aneurysms and pseudoaneurysms. J Vasc Surg.

[21] Pulli R, Dorigo W, Troisi N, Pratesi G, Innocenti AA, Pratesi C (2008). Surgical treatment of visceral artery aneurysms: a 25-year experience. J Vasc Surg.

[22] Luebke T, Heckenkamp J, Gawenda M, Beckurts KT, Lackner K, Brunkwall J (2007). Combined endovascular-open surgical procedure in a great hepatic artery aneurysm. Ann Vasc Surg.

[23] Popov P, Boskovic S, Sagic D, Radevic B, Ilijevski N, Nenezic D, Tasic N, Davidovic L, Radak D (2007). Treatment of visceral artery aneurysms: Retrospective study of 35 cases. Vasa.

[24] Cochennec F, Riga CV, Allaire E, Cheshire NJ, Hamady M, Jenkins MP, Kobeiter H, Wolfe JN, Becquemin JP, Gibbs RG (2011). Contemporary management of splanchnic and renal artery aneurysms: results of endovascular compared with open surgery from two European vascular centers. Eur J Vasc Endovasc Surg.

[25] Carr SC, Mahvi DM, Hoch JR, Archer CW, Turnipseed WD (2001). Visceral artery aneurysm rupture. J Vasc Surg.

